# Dietary fructose and risk of metabolic syndrome in Chinese residents aged 45 and above: results from the China National Nutrition and Health Survey

**DOI:** 10.1186/s12937-021-00739-9

**Published:** 2021-10-03

**Authors:** Shaojie Pang, Pengkun Song, Xueqian Sun, Wentao Qi, Chun Yang, Ge Song, Yong Wang, Jian Zhang

**Affiliations:** 1 Institute of Grain Quality and Nutrition Research, Academy of National Food and Strategic Reserves Administration, Beijing, 100037 People’s Republic of China; 2 National Institute for Nutrition and Health, Chinese Center for Disease Control and Prevention, Beijing, 100050 People’s Republic of China; 3 Research and Development center of Shandong Xiwang Sugar Co. Ltd, National Corn Deep Processing Industry Technology Innovation Center, Binzhou, People’s Republic of China; 4 Department of Nutrition and Food Hygiene, School of Public Health, Capital Medical University, Beijing, 100069 People’s Republic of China

**Keywords:** Dietary fructose, Metabolic syndrome, Chinese residents, Physical activity

## Abstract

**Background:**

A growing number of researches supported that dietary fructose was associated with most of the key features of metabolic syndrome (MetS). However, there was no related epidemiological studies among Chinese population, despite the sharp increase in MetS cases. This study explores the relationship between dietary fructose and MetS among Chinese residents aged 45 and above.

**Methods:**

A total of 25,528 participants (11,574 males and 13,954 females) were included in this nationwide representative cross-sectional study of China National Nutrition and Health Survey. Dietary fructose intake was assessed by 3-day 24-h dietary records. MetS was defined by the International Diabetes Federation and Chinese Diabetes Society criteria.

**Results:**

The consumption of dietary fructose was 11.6 g/day for urban residents and 7.6 g/day for rural residents. Fruits and vegetables as well as their products were the main sources of fructose intake. There was no association between dietary fructose intake and the odds of having MetS in both urban (*P* = 0.315) and rural residents (*P* = 0.230) after adjustment for confounding factors. Moreover, for urban residents participating physical activities, the odds of having MetS in the fourth quartiles (OR: 0.67; 95%CI: 0.52-0.87) was lower than that in the first quartile. In the sensitivity analysis, a significant reduction in the odds of having MetS was also found in the fourth quartiles (OR, 95%CI: 0.68, 0.51-0.90; 0.67, 0.49-0.91; 0.74, 0.56-0.99) compared with the first quartile when excluding smokers, alcohol users, and underweight/obesity, respectively. And there was no association between dietary fructose intake and the odds of having MetS after multivariate adjustment stratified by gender, smoking and alcohol use.

**Conclusions:**

Under the current dietary fructose intake status, there was no association between dietary fructose intake and the odds of having MetS among Chinese residents aged 45 and above. Physical activity and relatively low fructose intake may have a beneficial synergistic effect on MetS.

**Supplementary Information:**

The online version contains supplementary material available at 10.1186/s12937-021-00739-9.

## Introduction

Metabolic syndrome (MetS) refers to a series of cardio-metabolic risk factors leading to high risk for developing cardiovascular disease, type 2 diabetes, non-alcoholic fatty liver, and chronic kidney disease. Its symptoms include abdominal obesity, hyperglycemia, hypertension, and dyslipidemia [1, 2]. Over the past few decades, the prevalence of MetS has increased dramatically and has become one of the major public-health challenges in China and worldwide [2–4]. The overall standardized prevalence of MetS was 24.2% among Chinese adults and over 32% for those who aged 45 and above [5].

Increased sugar intake was widely recognized as a contributor to the worldwide epidemics of obesity, diabetes, and their associated cardio-metabolic risks [6]. Due to its unique set of biochemical, metabolic, and endocrine responses, fructose was usually regarded as the main negative factor in sugars and associated with all the key features of MetS [6, 7]. A series of systematic reviews and meta-analysis discussed the relationship between fructose and components of MetS. Some of them concluded that intakes of fructose were associated with increased risk of obesity, dyslipidemia, hypertension and cardio-metabolic syndrome [8–11]. However, some of them found that a certain dose of fructose had no adverse or even some positive effects on fasting glucose, blood pressure, and blood lipids [12–16]. Most of the studies included in these meta-analyses were interventional studies with high-dose fructose intake. In the “real world” study, a cross-sectional population-based research on Iranians reported that higher consumption of dietary fructose increased the risk of MetS, while no such association was found in the US population [17, 18]. Differences in dietary intake may be an important reason for the different results. Our previous research found that the average dietary fructose intake of Chinese residents aged 45 and above was 8.29 g/d, which is lower than that of Americans (48.07 g/d) and Iranians (46.50 g/d for male and 37.30 g/d for female) [19]. Up to now, there is no large epidemiological study to explore the relationship between dietary fructose and MetS under the current intake level among Chinese population although the prevalence of MetS has increased rapidly.

Based on data of nationally representative cross-sectional survey of China National Nutrition and Health Survey (CNNHS) in 2010-2012, this study aims to investigate the association between dietary fructose intake and MetS among Chinese residents aged 45 and above. Furthermore, we stratified the analysis based on the variables (gender, physical activity, smoking, and alcohol use) that might influence the odds of having MetS.

## Materials and method

### Study design and subjects

The nationwide representative cross-sectional study of CNNHS was conducted between 2010 to 2012 by Chinese Center for Disease Control and Prevention to assess the nutrition and health status of Chinese population. This survey covered all 31 provinces, autonomous regions, and municipalities directly under the Chinese central government (excluding Taiwan, Hong Kong, and Macao). A stratified multistage random cluster sampling method was conducted at 150 surveys sites of 4 types including 34 large cities, 41 small-to-medium cities, 45 general rural areas and 30 poor rural areas. The survey procedure has been described in detail elsewhere [20]. All participants were supposed to undergo three consecutive 24-h dietary records combined with food weighting, survey questionnaires, physical examination, and fasting blood collection.

In this study, we included participants aged 45 and above with complete demographic information, medical history, lifestyle factors and dietary intake data. We excluded those with implausible energy intakes (< 800 kcal/day or > 4800 kcal/day for male and < 500 kcal/day or > 4000 kcal/day for female). Eventually, a total of 25,528 participants (11,574 males and 13,954 females) were included [20].

This survey was ethically approved by the Ethical Committee of the National Institute for Nutrition and Food Safety, Chinese Center for Disease Control and Prevention (2013-018). Written informed consent was obtained from all participants.

### Data collection and definition

Data were collected by trained health workers or nurses in health examination centers from local health stations or community clinics according to a standard protocol. Questionnaires including demographic information, medical history, and lifestyle factors, were conducted by trained interviewers. Marital status was categorized into three statues (single, married, divorced/widowed). Education levels were classified into primary schools or below, junior high school, senior high school, and college or above. Smoking was defined as “having smoked 100 cigarettes during lifetime” and “current smoking”. Alcohol was referred to as “alcohol intake more than once per month during the past 12 months”. Physical activity was defined as “moderate physical activity for more than 10 minutes at least once per week” [21]. According to the grading standards of national residents’ net income levels by National Bureau of Statistics in 2009, high income was defined as “≥ 20000 per person per year for urban residents or ≥ 10000 for rural”, middle income as “15000 ~ 19999 for urban or 5000 ~ 9999 for rural”, and low income as “< 15000 for urban or < 5000 for rural”.

Height, weight, waist circumference (WC) and blood pressure (BP) were measured in the morning with standardized procedures. Height was measured in bare feet to the nearest 0.1 cm. Weight was measured in standing position and light clothing to the nearest 0.1 kg. Body mass index (BMI) was calculated with weight (kg) being divided by height (m) squared. WC was measured in standing position between the lower edge of the costal arch and the upper edge of the iliac crest. BP levels were measured for 3 times in succession with 1-min interval between the measurements, with a standard mercury sphygmomanometer at the nondominant arm in setting position after 5 min of rest. Systolic blood pressure (SBP) was measured at the first appearance of a pulse sound (Korotkoff phase 1) and diastolic blood pressure (DBP) at the disappearance of the pulse sound (Korotkoff phase 5). The mean of the three measurements was used for analysis.

### Dietary data and assessment of dietary fructose intake

Data as individual dietary records and household food consumptions were collected over three consecutive days. Individual dietary data including all foods consumed at home and away from home (type, amounts, type of meal, and place of consumption) were collected by trained dietary investigators. Weighting method was used to assess household food consumptions, which included all foods and condiments. The Chinese Food Composition was used to calculate the amount of energy, protein, fat, carbohydrate, and fiber contained in each food consumed by individuals every day.

Since there was no fructose content data in some of the Chinese Food Composition (1460 food items), we used fructose content data of the American Food Composition Database (2183 food items) and Chinese Sugar Content Database in Pre-packaged Foods (363 food items) to assign the value of fructose content for each food item [22, 23]. The principle of food fructose content assignment was described in detail in our previous study [19].

Total dietary fructose was composed of free-fructose and bound-fructose. Free-fructose intake for each person was calculated from dietary supplement and natural food intake. Bound-fructose intake for each person was calculated by using one-half of dietary total sucrose based on the molecular compositing of sucrose. All forms of fructose including food-fructose and food-sucrose were added to obtain the total fructose intake of each participant based on the food composition database [17].

All foods listed in the food-grouping system used in Chinese Food Composition Table were divided into 13 categories according to the major ingredients: grain and grain products; fruits and fruit products; vegetables and vegetable products; milk and milk products; meat, poultry, fish, and related products; eggs and egg products; legumes and legume products; nuts, seed, and related products; sugars and sweets; nonalcoholic beverages; alcoholic beverages; snacks; and miscellaneous foods.

### Anthropomorphic and blood biochemical methods

Blood samples were collected by trained nurses from all participants undergoing an overnight fast for at least 10 h. Samples were centrifuged at 1500 rpm for 10 min after being left standing for 30 to 60 min. The centrifuged serum sample were transported to the central laboratory of the National Institute for Nutrition and Health and stored at − 80 degrees centigrade. Procedure, processing, and determination for the blood collection were standardized. Fasting plasma glucose (FPG), total cholesterol (TC), high-density lipoprotein cholesterol (HDL-C) and triglycerides (TG) were measured by a Hitachi automatic biochemical analyzer with reagents from Wako Pure Chemical Industries, Ltd. (Tokyo, Japan).

### Definition of MetS

According to the recommendation from the International Diabetes Federation and Chinese Diabetes Society criteria [24, 25], a person who met three or more of the following five criteria were diagnosed as MetS: (1) abdominal obesity (WC ≥ 90 cm in male or ≥ 85 cm in female); (2) hyperglycemia (FPG ≥ 6.1 mmol/L or diagnosed diabetes); (3) hypertension (SBP ≥ 130 mmHg or DBP ≥ 85 mmHg or diagnosed hypertension); (4) TG ≥ 1.70 mmol/L; (5) HDL-C < 1.04 mmol/L.

### Statistical analysis

Data were collected with specialized software. Data cleaning and statistical analyses were performed by using SAS version 9.4 (SAS Institute Inc., Cary, NC, USA). Due to the differences of total dietary fructose intake and the prevalence of MetS between urban and rural areas, analyses were performed separately in regard with urban and rural samples. Categorical variables were presented as percentage and examined with a chi-square test. Continuous variables with normal distribution were presented as mean (95%CI) and were compared across groups with z test. Skewed distribution variables were indicated with quartiles and were examined by non-parametric statistical hypothesis test. The Cochran and Mantel-Haenszel test were used to analyze the characteristics of normal and MetS in urban and rural areas. Univariate and multivariable-adjusted logistic regression were performed to explore the association between dietary fructose intake and risk of MetS. The first quartile of total dietary fructose intake was set as the reference. Three models were involved in this study: model 1 did not adjust any variables; model 2 adjusted for gender, age, education, marital status, smoking, alcohol, physical activity, income, energy, protein, fat, carbohydrate, fiber, and TC; model 3 adjusted for all variables in model 2 plus BMI. Odds ratios (OR) and 95%CI were measured. A value of *P* < 0.05 was considered statistically significant.

## Results

### Basic characteristics of the study population

A total of 25,528 participants were included in the study with an average age of 59.1 years old in which 13,067 (44.1% males) were urban residents and 12,461 (46.6% males) were rural residents. And significant differences were found between urban and rural participants regarding age, gender, marital status, education, smoking, alcohol, physical activity, and income. BMI, WC, TC, TG, and FPG were higher in urban participants than that in rural participants (*P* < 0.001), whereas HDL-C was the opposite (*P* < 0.001, Table 1). Table 2 showed that the prevalence of MetS in urban areas was32.4% which was higher than that in rural with 24.7% (*P* < 0.001).

**Table 1:** Basic characteristics of the study population in urban and rural

	Total	Urban	Rural	*p*-Value
N	25,528	13,067	12,461	
Age, years	59.1 (59.0, 59.2)	59.7 (59.5, 59.8)	58.5 (58.3, 58.7)	< 0.001
Gender, n (%)				
Male	11,574 (45.3)	5762 (44.1)	5812 (46.6)	
Female	13,954 (54.7)	7305 (55.9)	6649 (53.4)	
Marital status, n (%)				0.011
Single	181 (0.7)	84 (0.6)	97 (0.8)	
Married	23,093 (90.5)	11,767 (90.1)	11,326 (90.9)	
Divorced or Widowed	2254 (8.8)	1216 (9.3)	1038 (8.3)	
Education, n (%)				< 0.001
Primary schools or below	12,407 (48.6)	4426 (33.9)	7981 (64.1)	
Junior high school	7980 (31.3)	4556 (34.9)	3424 (27.5)	
Senior high school	3963 (15.5)	2975 (22.8)	988 (7.9)	
College or above	1178 (4.6)	1110 (8.5)	68 (0.6)	
Smoking, n (%)				< 0.001
Ever/Never	6830 (26.8)	3065 (23.5)	3765 (30.2)	
Current	18,698 (73.2)	10,002 (76.5)	8696 (69.8)	
Alcohol, n (%)				< 0.001
Ever/Never	8169 (32.0)	4189 (32.1)	3980 (31.9)	
Current	17,359 (68.0)	8878 (67.9)	8481 (68.1)	
Physical activity, n (%)				< 0.001
Yes	4012 (15.7)	3309 (25.3)	703 (5.6)	
No	21,516 (84.3)	9758 (74.7)	11,758 (94.4)	
Income, n (%)				< 0.001
Low	11,940 (46.8)	7024 (53.8)	4916 (39.5)	
Middle	5202 (20.4)	1833 (14.0)	3369 (27.0)	
High	7276 (28.5)	3456 (26.5)	3820 (30.7)	
Unanswered	1110 (4.4)	754 (5.8)	356 (2.9)	
BMI, kg/m^2^	24.1 (24.1, 24.2)	24.6 (24.6, 24.7)	23.6 (23.6, 23.7)	< 0.001
WC, cm	82.9 (82.7, 83.0)	84.2 (84.0, 84.3)	81.5 (81.3, 81.6)	< 0.001
SBP, mmHg	130.9 (130.6, 131.1)	130.7 (130.4, 131.1)	131.0 (130.6, 131.4)	0.640
DBP, mmHg	81.0 (80.8, 81.1)	80.9 (80.7, 81.1)	81.1 (81.9, 81.3)	0.929
TC, mmol/L	4.79 (4.78, 4.80)	4.89 (4.87, 4.90)	4.70 (4.68, 4.72)	< 0.001
HDL-C, mmol/L	1.19 (1.19, 1.20)	1.18 (1.18, 1.19)	1.20 (1.20, 1.21)	< 0.001
TG, mmol/L	1.50 (1.49, 1.51)	1.55 (1.53, 1.57)	1.44 (1.42, 1.46)	< 0.001
FPG, mmol/L	5.52 (5.50, 5.54)	5.67 (5.64, 5.69)	5.36 (5.34, 5.39)	< 0.001

Mean value (95% confidence interval) or n (%) were shown; *BMI* body mass index, *WC* waist circumference, *SBP* systolic blood pressure, *DBP* diastolic blood pressure, *TC* total cholesterol, *HDL-C* high-density lipoprotein cholesterol, *TG* triglyceride, *FPG* fasting plasma glucose

**Table 2:** Basic characteristics of normal and MetS in urban and rural

	Urban		Rural		*p*-Value
	Normal	MetS	Normal	MetS	
Case, n (%)	8830 (67.6)	4237 (32.4)	9388 (75.3)	3073 (24.7)	< 0.001
Age, n (%)					< 0.001
45-59 years	5078 (57.5)	2004 (47.3)	5598 (59.6)	1702 (55.4)	
60-74 years	3353 (38.0)	1997 (47.1)	3420 (36.4)	1247 (40.6)	
75- years	399 (4.5)	236 (5.6)	370 (3.9)	124 (4.0)	
Gender, n (%)					0.248
Male	3788 (42.9)	1974 (46.6)	4526 (48.2)	1286 (41.9)	
Female	5042 (57.1)	2263 (53.4)	4862 (51.8)	1787 (58.2)	
Marital status, n (%)					0.246
Single	63 (0.7)	21 (0.5)	88 (0.9)	9 (0.3)	
Married	7956 (90.1)	3811 (90.0)	5811 (90.7)	2815 (91.6)	
Divorced or Widowed	811 (9.2)	405 (9.6)	789 (8.4)	249 (8.1)	
Education, n (%)					< 0.001
Primary schools or below	3022 (34.2)	1404 (33.1)	6092 (64.9)	1889 (61.5)	
Junior high school	3062 (34.7)	1494 (35.3)	2546 (27.1)	878 (28.6)	
Senior high school	2035 (23.1)	940 (22.2)	709 (7.6)	279 (9.1)	
College or above	711 (8.1)	399 (9.4)	41 (0.4)	27 (0.9)	
Smoking, n (%)					< 0.001
Ever/Never	6725 (76.2)	3277 (77.3)	6248 (66.6)	2233 (72.7)	
Current	2105 (23.8)	960 (22.7)	3140 (33.4)	840 (27.3)	
Alcohol, n (%)					< 0.001
Ever/Never	5926 (67.1)	2952 (69.7)	6374 (67.9)	2322 (75.6)	
Current	2904 (32.9)	1285 (30.3)	3014 (32.1)	751 (24.4)	
Physical activity, n (%)					< 0.001
Yes	2149 (24.3)	1160 (27.4)	460 (4.9)	243 (7.9)	
No	6681 (75.7)	3077 (72.6)	8928 (95.1)	2830 (92.1)	
Income, n (%)					0.004
Low	4835 (54.8)	2189 (51.7)	3713 (39.6)	1203 (39.2)	
Middle	1195 (13.5)	638 (15.1)	2594 (27.6)	775 (25.2)	
High	2273 (25.7)	1183 (27.9)	2819 (30.0)	1001 (32.6)	
Unanswered	527 (6.0)	227 (5.4)	262 (2.8)	94 (3.1)	

### Dietary fructose intake level and food sources

Table 3 shows the basic characteristics of dietary fructose and nutrients with mean and quartiles. The average daily total dietary fructose intake for urban residents was 11.6 g and 7.6 g for rural residents. Total dietary fructose intake level was significantly higher in urban residents than that in rural residents, including both free-fructose and bound-fructose levels (*P* < 0.001).Protein and fat intake were significantly higher in urban residents while energy and carbohydrate were higher in rural residents (*P* < 0.001).

**Table 3:** Intake status of dietary fructose and nutrients in urban and rural residents

	City				Rural				*p*-Value
	Mean	P_25th_	Median	P_75th_	Mean	P_25th_	Median	P_75th_	
Total Fructose, g/d	11.6	4.8	8.3	14.5	7.6	3.4	5.3	8.8	< 0.001
Free-Fructose, g/d	6.7	2.2	4.2	8.4	4.6	1.6	2.7	5.1	< 0.001
Bound-Fructose, g/d	4.9	2.4	3.7	6.0	3.1	1.6	2.4	3.7	< 0.001
Energy, kcal/d	1887	1454	1780	2204	2159	1648	2041	2547	< 0.001
Protein, g/d	61.2	44.8	56.6	72.5	59.3	43.6	55.4	71.2	< 0.001
Fat, g/d	76.1	50.2	69.2	94.1	70.8	44.0	64.0	90.2	< 0.001
Carbohydrate, g/d	240.3	178.0	224.9	284.3	322.7	238.7	299.4	378.7	< 0.001
Fiber, g/d	10.7	6.7	9.2	12.9	10.5	6.7	9.2	12.5	0.957

**Fig. 1:**
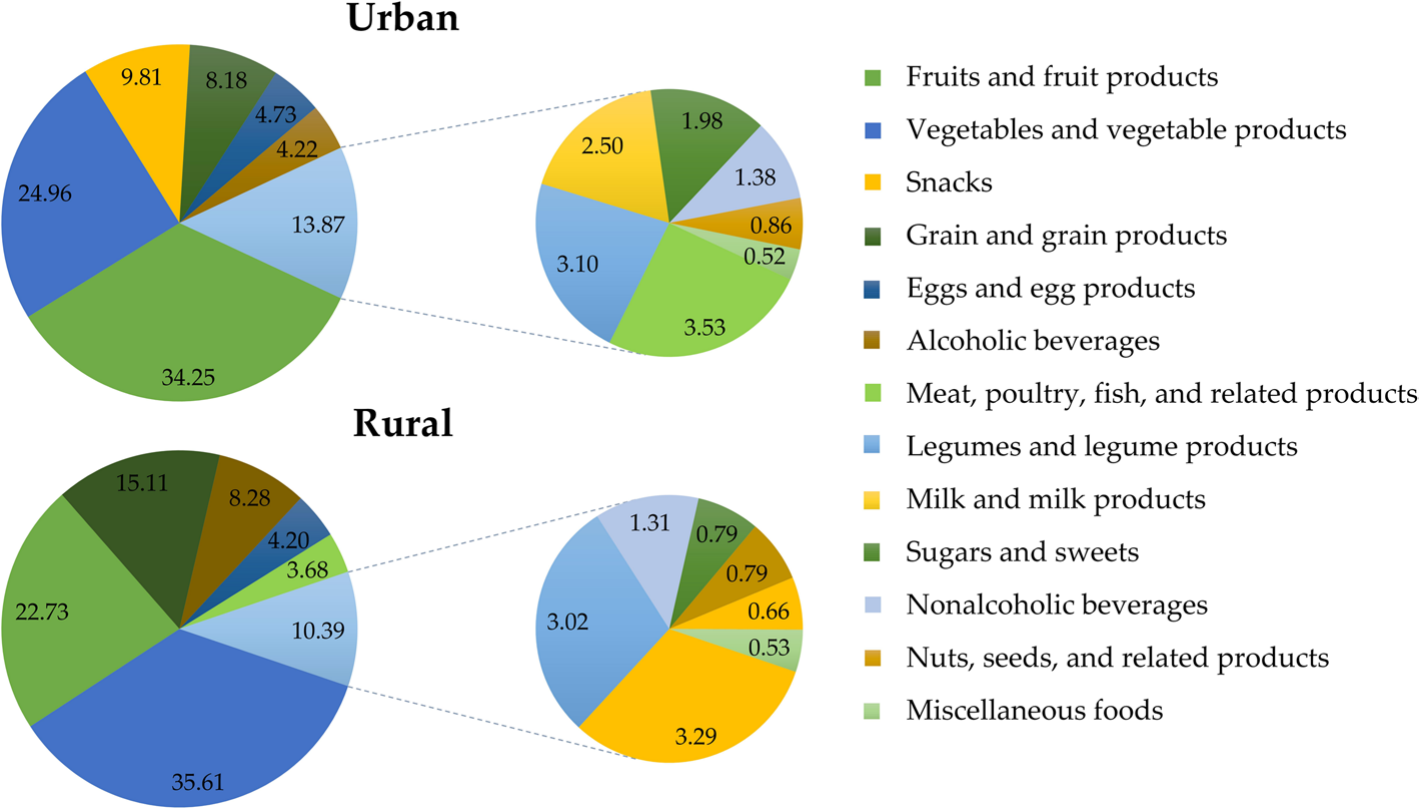
Distribution of dietary fructose from various foods in urban and rural residents. The smaller circle represents a part of the larger circle. The proportion of small circle in great circle for urban was 13.87 and 10.39% for rural

We further investigated the food sources of dietary fructose. Fruits and fruit products, vegetables and vegetable products, and snacks were the top three food sources for dietary fructose among urban residents accounting 69.02% for the total dietary fructose intake, while vegetables and vegetable products, Fruits and fruit products, and grain and grain products were the top three food sources for dietary fructose in rural residents contributing 73.45% to the total dietary fructose intake (Fig. 1). Food sources of total dietary fructose in urban and rural areas with mean and quartiles were shown in Table S1.

### The association between dietary fructose intake and the odds of having MetS

For urban residents, in addition to FPG, there were significant differences in WC, SBP, DBP, TG and HDL-C between the quartiles. The prevalence of MetS was higher in the third quartile than that in the first and forth quartiles (*P* < 0.05). For rural residents, we found significant differences in FPG, SBP, DBP, TG, and HDL-C between the quartiles except for WC. There was no significant difference in the prevalence of MetS between quartiles (Table 4).

**Table 4:** Basic characteristics of components of MetS by the quartiles of dietary fructose intake

	Q1	Q2	Q3	Q4	*p*-Value
**Urban**	3266	3268	3266	3267	
Dietary fructose	3.4 (3.4, 3.4)	6.4 (6.4, 6.4)	11.1 (11.1, 11.1)	25.6 (25.1, 26.2)	
WC, cm	83.5 (83.2, 83.8)	84.5 (84.1, 84.8)^1^	84.4 (84.1, 84.8) ^1^	84.3 (83.9, 84.6) ^1^	< 0.001
FPG, mmol/L	5.68 (5.63, 5.73)	5.72 (5.67, 5.78)	5.64 (5.59, 5.69)	5.62 (5.57, 5.67)	0.081
SBP, mmHg	131.8 (131.1, 132.5)	132.0 (131.3, 132.7)	130.3 (129.7, 131.0) ^1, 2^	128.8 (128.2, 129.4) ^1–3^	< 0.001
DBP, mmHg	81.0 (80.6, 81.4)	81.5 (81.2, 81.9)	80.8 (80.5, 81.2)	80.2 (79.9, 80.6) ^1, 2^	< 0.001
TG, mmol/L	1.49 (1.46, 1.53)	1.56 (1.53, 1.60) ^1^	1.59 (1.55, 1.62) ^1^	1.56 (1.53, 1.60)	0.002
HDL-C, mmol/L	1.20 (1.19, 1.22)	1.19 (1.18, 1.20)	1.18 (1.16, 1.18) ^1^	1.17 (1.16, 1.18) ^1^	< 0.000
MetS, n (%)	1019 (31.2)	1083 (33.1)	1109 (34.0)^1^	1027 (31.4)^3^	0.048
**Rural**	3115	3116	3115	3115	
Dietary fructose	2.5 (2.5, 2.5)	4.3 (4.3, 4.3)	6.8 (6.8, 6.8)	16.8 (16.4, 17.2)	
WC, cm	81.1 (80.7, 81.4)	81.5 (81.1, 81.8)	81.7 (81.3, 82.1)	81.6 (81.3, 81.9)	0.087
FPG, mmol/L	5.37 (5.32, 5.42)	5.44 (5.39, 5.49)	5.32 (5.28, 5.37)^2^	5.32 (5.27, 5.37) ^2^	0.001
SBP, mmHg	132.6 (131.9, 133.4)	131.2 (130.5, 131.9)^1^	130.9 (130.2, 131.7) ^1^	129.3 (128.5, 130.0) ^1–3^	< 0.001
DBP, mmHg	81.6 (81.2, 82.0)	81.0 (80.6, 81.4)	81.0 (80.6, 81.4)	80.7 (80.3, 81.1) ^1^	0.017
TG, mmol/L	1.39 (1.35, 1.42)	1.43 (1.39, 1.47)	1.43 (1.39, 1.47)	1.52 (1.48, 1.57) ^1–3^	< 0.001
HDL-C, mmol/L	1.22 (1.21, 1.24)	1.22 (1.21, 1.23)	1.20 (1.18, 1.21) ^1, 2^	1.18 (1.16, 1.19) ^1, 2^	< 0.001
MetS, n (%)	764 (24.5)	772 (24.8)	805 (25.8)	732 (23.5)	0.199

^1^: compared with Q1, *p* < 0.05^2^;: compared with Q2, *p* < 0.05^3^;: compared with Q3, *p* < 0.05

Compared with the first quartile, the odds of MetS was higher in the third quartile among urban residents (OR: 1.13; 95%CI: 1.02-1.26). After adjusting confounding factors (gender, age, education, marital status, smoking, alcohol, physical activity, income, energy, protein, fat, carbohydrate, TC and BMI), no statistical significance was found. For rural residents, regardless of adjustments made for confounding factors, no association between dietary fructose intake and the odds of having MetS was found (Table 5).

**Table 5:** The association between dietary fructose intake and the odds of having MetS

	Dietary fructose intake				*p*-Value
	Q1	Q2	Q3	Q4	
**Urban**					
Model 1	1.00	1.09 (0.98, 1.21)	1.13 (1.02, 1.26)	1.01 (0.91, 1.12)	**0.048**
Model 2	1.00	1.08 (0.97, 1.20)	1.10 (0.99, 1.23)	0.97 (0.86, 1.09)	**0.058**
Model 3	1.00	1.03 (0.92, 1.17)	1.08 (0.96, 1.22)	0.97 (0.85, 1.11)	0.336
**Rural**					
Model 1	1.00	1.01 (0.90, 1.14)	1.07 (0.96, 1.20)	0.95 (0.84, 1.06)	0.199
Model 2	1.00	1.03 (0.92, 1.16)	1.13 (1.00, 1.27)	1.03 (0.90, 1.17)	0.213
Model 3	1.00	1.03 (0.90, 1.18)	1.14 (0.99, 1.31)	1.03 (0.89, 1.19)	0.246

Model 1: crude; Model 2: adjusted gender, age, education, marital status, smoking, alcohol, physical activity, income, energy, protein, fat, carbohydrate, fiber, TC; Model 3: model 2 plus BMI

### Stratified analysis of the association between dietary fructose intake and risk of MetS

We further analyzed the association between dietary fructose intake and the odds of having MetS stratified by gender, physical activity, smoking, and alcohol use. For urban residents with physical activities, the prevalence and the odds of MetS were both lower with the increase of the quartile levels of dietary fructose intake (*P* < 0.001). Compared with the first quartile, the odds of having MetS in the fourth quartile (OR: 0.67; 95%CI: 0.52-0.87) was lower after adjustment for confounding factors (Table 6). In the sensitivity analysis, we also found a significant reduction in the odds of having MetS in the fourth quartile (OR, 95%CI: 0.68, 0.51-0.90; 0.67, 0.49-0.91; 0.74, 0.56-0.99) compared with the first quartile when excluding smokers, alcohol users, and BMI < 18.5 or BMI ≥ 28, respectively (Table 7). For urban residents with no physical activities, the prevalence of MetS increased with the increase of dietary fructose intake (*P* = 0.008). There was no significant difference for the relationship between dietary fructose intake and the odds of having MetS after multivariate adjustment, as was the case in the sensitivity analysis (Tables 6, 7).

**Table 6:** Stratified analysis of the association between dietary fructose intake and the odds of having MetS by physical activity in urban residents

	Dietary fructose intake				*p*-Value
	Q1	Q2	Q3	Q4	
Physical activity					
MetS, n (%)	233 (42.1)	236 (36.2)	324 (35.9)	367 (30.6)	**< 0.001**
Model 1	1.00	0.78 (0.62, 0.99)	0.77 (0.62, 0.96)	0.61 (0.49, 0.75)	**< 0.001**
Model 2	1.00	0.79 (0.62, 1.00)	0.79 (0.63, 0.99)	0.63 (0.50, 0.80)	**0.002**
Model 3	1.00	0.79 (0.61, 1.03)	0.82 (0.63, 1.05)	0.67 (0.52, 0.87)	**0.026**
Non-physical activity					
MetS, n (%)	786 (29.0)	846 (32.3)	785 (33.2)	660 (32.0)	**0.007**
Model 1	1.00	1.17 (1.04, 1.32)	1.22 (1.08, 1.37)	1.15 (1.02, 1.30)	**0.007**
Model 2	1.00	1.15 (1.02, 1.30)	1.18 (1.04, 1.34)	1.09 (0.95, 1.26)	**0.046**
Model 3	1.00	1.11 (0.97, 1.27)	1.15 (1.00, 1.33)	1.09 (0.93, 1.27)	0.252

Model 1: crude; Model 2: adjusted gender, age, education, marital status, smoking, alcohol, income, energy, protein, fat, carbohydrate, fiber, TC; Model 3: model 2 plus BMI

**Table 7:** Sensitivity analysis of the association between dietary fructose intake and the odds of having MetS by physical activity in urban residents

	Dietary fructose intake				*p*-Value
	Q1	Q2	Q3	Q4	
Physical activity					
MetS, n (%)^a^	188 (41.3)	185 (34.9)	256 (35.1)	313 (30.1)	**< 0.001**
Model 1^a^	1.00	0.76 (0.59, 0.99)	0.77 (0.60, 0.98)	0.61 (0.49, 0.77)	**< 0.001**
Model 2^a^	1.00	0.77 (0.59, 1.01)	0.78 (0.60, 1.00)	0.62 (0.48, 0.80)	**0.004**
Model 3^a^	1.00	0.77 (0.57, 1.03)	0.82 (0.62, 1.08)	0.68 (0.51, 0.90)	**0.042**
MetS, n (%)^b^	168 (42.5)	166 (37.8)	207 (34.2)	230 (30.1)	**< 0.001**
Model 1^b^	1.00	0.82 (0.62, 1.08)	0.70 (0.54, 0.91)	0.58 (0.45, 0.75)	**< 0.001**
Model 2^b^	1.00	0.84 (0.63, 1.11)	0.71 (0.54, 0.93)	0.59 (0.44, 0.78)	**0.002**
Model 3^b^	1.00	0.87 (0.64, 1.19)	0.76 (0.56, 1.00)	0.67 (0.49, 0.91)	**0.043**
MetS, n (%)^c^	147 (33.2)	166 (30.8)	234 (31.0)	268 (26.0)	**0.017**
Model 1^c^	1.00	0.90 (0.69, 1.17)	0.90 (0.70, 1.16)	0.71 (0.56, 0.90)	**0.017**
Model 2^c^	1.00	0.84 (0.63, 1.11)	0.71 (0.54, 0.93)	0.59 (0.44, 0.78)	**0.002**
Model 3^c^	1.00	0.89 (0.66, 1.20)	0.92 (0.70, 1.23)	0.74 (0.56, 0.99)	**0.151**
Non- physical activity					
MetS, n (%)^a^	602 (29.6)	650 (34.36)	590 (33.50)	493 (31.58)	**0.008**
Model 1^a^	1.00	1.25 (1.09, 1.42)	1.20 (1.05, 1.38)	1.10 (0.95, 1.27)	**0.008**
Model 2^a^	1.00	1.22 (1.06, 1.40)	1.17 (1.01, 1.35)	1.06 (0.90, 1.24)	**0.021**
Model 3^a^	1.00	1.15 (0.99, 1.35)	1.15 (0.98, 1.35)	1.07 (0.89, 1.28)	0.223
MetS, n (%)^b^	602 (31.1)	620 (34.0)	542 (33.8)	417 (31.8)	0.182
Model 1^b^	1.00	1.14 (0.99, 1.31)	1.13 (0.98, 1.30)	1.03 (0.89, 1.20)	0.182
Model 2^b^	1.00	1.12 (0.97, 1.29)	1.09 (0.94, 1.27)	0.98 (0.83, 1.16)	0.221
Model 3^b^	1.00	1.07 (0.92, 1.25)	1.05 (0.89, 1.25)	0.95 (0.78, 1.15)	0.503
MetS, n (%)^c^	541 (24.3)	572 (27.0)	534 (27.6)	450 (26.3)	0.073
Model 1^c^	1.00	1.16 (1.01, 1.33)	1.19 (1.03, 1.37)	1.12 (0.97, 1.29)	0.073
Model 2^c^	1.00	1.12 (0.97, 1.29)	1.09 (0.94, 1.27)	0.98 (0.83, 1.16)	0.221
Model 3^c^	1.00	1.12 (0.96, 1.31)	1.14 (0.97, 1.33)	1.06 (0.89, 1.26)	0.354

Model 1: crude; Model 2: adjusted gender, age, education, marital status, smoking, alcohol, income, energy, protein, fat, carbohydrate, fiber, TC; Model 3: model 2 plus BMI. ^a^excluded smokers; ^b^ excluded alcohol; ^c^ excluded BMI < 18.5, and BMI ≥ 28

There was no association between dietary fructose intake and the odds of having MetS for rural residents regardless of physical activities (Table S2). When stratified by gender, the prevalence of MetS was the lowest in the first quartile of dietary fructose intake for urban males, but in the fourth quartile for urban females (Table S3). And there was no association between dietary fructose intake and the odds of having MetS after multivariate adjustment stratified by gender, smoking and alcohol (Tables S3, S4, S5).

## Discussion

In this nationwide representative cross-sectional study, we discussed the association between dietary fructose intake and the odds of having MetS among Chinese residents aged 45 and above. The consumption of dietary fructose for urban residents was 11.6 g/day and for rural residents was 7.6 g/day. Under the current dietary fructose intake status, we did not find associations between dietary fructose intake and the odds of having MetS in both urban and rural residents. However, there was a significant inverse association between dietary fructose intake and MetS for urban residents with physical activities.

A large number of researches suggested that fructose was a culprit in the occurrence of MetS through several metabolic pathways, such as increasing hepatic de novo lipogenesis in the liver [26], depleting ATP stores which results in increasing generation of uric acid via purine pathway [27, 28], affecting on plasma lipids, lipoprotein, and apolipoproteins [29, 30], and interacting with host-gastrointestinal microbe interactions [31, 32]. However, there were still disputes between mechanism studies and population epidemiological studies. According to the systematic reviews and meta-analysis, high doses of fructose (≥100 g/day) increased serum TG concentration [10, 33], low to middle doses of fructose (0 ~ 90 g/day) had a benefit effect in HbA1c [14, 34]. But fructose did not increase the risk of hypertension and type 2 diabetes [13, 35], also, it did not affect serum HDL-C concentration [33] and cause weight gain when it replaced other carbohydrate in diets to provide similar calories [36].

In present study, we did not find an association between dietary fructose intake and the odds of having MetS among Chinese residents aged 45 and above. The results of this study were consistent to the study from the NHANES 1999-2006 which showed that ordinary fructose consumption (approximately 37% of total sugars and 9% of daily energy in the US population) had no association with the odds of having MetS [18]. Both of the two studies were population-based cross-sectional studies. However, a systematic review and meta-analysis discussing the association of fructose consumption and components of MetS reported that fructose consumption was positively associated with FPG, TG and SBP, and negatively associated with HDL-C [9]. We assumed several reasons for the difference. On the one hand, the fructose sources were different. Food sources of fructose in this meta-analysis were from industrialized foods. In our study, however, fruits and fruit products, vegetables and vegetable products were the most dominant food sources, accounting for more than 50% of dietary fructose. One study reported that most food sources of dietary fructose (especially fruits) did not have a harmful effect on indicators of health (HbA1c, fasting insulin), but several food sources of fructose (especially sugars-sweetened beverages) adding excessive energy to diets showed negative effects [37]. On the other hand, the fructose intake was different. Fructose provided at least 15% of daily energy requirements in the 15 studies included in this meta-analysis. In our study, however, the average dietary fructose intakes for urban and rural residents was 11.6 g/day and 7.6 g/day, respectively. They contributed less than 3% of energy requirements. Several systematic reviews reported that a continuous exposure to high fructose intake may have adverse health effects [38, 39]. The previous study has shown that the percentage of total calories from added sugar containing food of Chinese residents in 2010-2010 was 9.09%, which was under the recommended limits (10%) of WHO [40, 41]. In addition, some researchers argued that the before-after design used by the authors, the lack of adjustment for energy as an important confounding variable, and unclear statistical methods all of these render their results as uninterpretable. Under calorie-matched conditions, this systematic review and meta-analysis cannot infer that fructose uniquely affects most components of MetS [42]. In this study, we not only adjusted the confounding factors, including energy, but also stratified analyzed the variables (gender, physical activity, smoking, and alcohol use) that might influence the odds of having MetS.

Interestingly, we found that the odds of MetS was lower with the increase of the quartile levels of dietary fructose intake for the urban residents with physical activities. In recent years, a growing number of researches supported the idea that physical activities might play a role of modulator for fructose’s health effects [38, 39, 43–45]. Fructose was generally processed in splanchnic organs (small bowel, liver, kidneys) and turned into glucose, lactate, and fatty acids, which serve as metabolic energy substrates in extra-splanchnic organs and tissues [38]. As fructose uptake and fructolysis were unregulated processes, the amount of metabolic energy substrates was proportionate to fructose intake [43]. For sedentary subjects, high fructose intake caused an overflow of metabolic energy substrates which resulted in increased gluconeogenesis, de novo lipogenesis, and triglyceride-rich lipoprotein secretion in the liver [43]. In contrast, for physically active subjects, a high fructose intake will be accompanied with high energy expenditure, in this way fructose would be mainly metabolized into glucose and lactate which can be readily oxidized to support ATP synthesis and result in a net lactate release from splanchnic organs (mostly the liver) to the working muscle [43]. This ‘reverse Cori cycle’ may be advantageous to improve performance by acting on central fatigue and/or alter metabolic regulation [44, 45]. An animal study had shown that the naked mole-rat can resist hypoxia and acidosis by increasing fructolysis [46]. In our study, dietary fructose intake in the fourth quartile of urban and rural residents was 25.6 g/day and 16.8 g/day, respectively, both of which were in relatively low dosages. A series of systematic reviews and meta-analyses have reported that small doses of fructose, or fructose in substitution for glucose or sucrose, may have beneficial effects or not any adverse effects on the components of the MetS [13–16, 34, 47, 48]. Based on the above points, we suggested that physical activities and relatively low fructose intake may have a beneficial synergistic effect on MetS.

Several limitations should be considered in the present study. First, this cross-sectional study has a natural disadvantage to address causal relationship between dietary fructose intake and MetS. Second, fructose additionally supplied was not distinguished in this study. In previous studies, the intake status of additional fructose and its relationship with metabolic disease were the focus of attention. However, the consumption of added fructose was very low in our study population. Third, the accuracy of dietary records was limited for the faint recalling of the participants’ and the specificity with which the reported foods were mapped in the dietary recall records. To minimize this situation, all the interviewers had completed a strict training program with detailed methodologies on administration of the dietary questionnaire. Forth, three consecutive 24-h dietary records may not enough to reflect long-term dietary habits. More high-quality cohort studies and randomized controlled trials were still needed to evaluate the association between dietary fructose intake and the risk of MetS.

## Conclusions

To our knowledge, the present study fills a gap by firstly discussing the association between dietary fructose and the odds of having MetS among Chinese residents aged 45 and above. Fruits and fruit products, vegetables and vegetable products were the main food sources, and the dietary fructose intake was relatively low. Under the current dietary fructose intake status, there was no association between dietary fructose intake and the odds of having MetS in both urban and rural residents. Interestingly, there was a significant inverse association between dietary fructose intake and MetS for urban residents who participating in physical activity. Our results indicated that physical activity and relatively low fructose intake may have an advantageous synergistic effect on MetS.

## Supplementary Information

**Additional file 1: Table S1.** Food sources of dietary fructose in urban and rural residents (g/d). **Table S2.** Stratified analysis of the association between dietary fructose intake and the odds of having MetS by physical activity in rural area. **Table S3.** Stratified analysis of the association between dietary fructose intake and the odds of having MetS by gender. **Table S4.** Stratified analysis of the association between dietary fructose intake and the odds of having MetS by smoking. **Table S5.** Stratified analysis of the association between dietary fructose intake and the odds of having MetS by alcohol use.

## Data Availability

Not applicable.

## Abbreviations

MetS: Metabolic syndrome
CNNHS: China National Nutrition and Health Survey
WC: Waist circumference
BMI: Body mass index
SBP: Systolic blood pressure
DBP: Diastolic blood pressure
FPG: Fasting plasma glucose
TC: Total cholesterol
HDL-C: High-density lipoprotein cholesterol
TG: Triglycerides
