# Supplementary material for: Dietary fructose and risk of metabolic syndrome in Chinese residents aged 45 and above: results from the China National Nutrition and Health Survey
Source: Nutr J. 2021 Oct 3;20:83. doi: 10.1186/s12937-021-00739-9 (PMC8489071; doi:10.1186/s12937-021-00739-9)
Supplement: Supplementary file 1 — Additional file 1: Table S1. Food sources of dietary fructose in urban and rural residents (g/d). Table S2. Stratified analysis of the association between dietary fructose intake and the odds of having MetS by physical activity in rural area. Table S3. Stratified analysis of the association between dietary fructose intake and the odds of having MetS by gender. Table S4. Stratified analysis of the association between dietary fructose intake and the odds of having MetS by smoking. Table S5. Stratified analysis of the association between dietary fructose intake and the odds of having MetS by alcohol use. [file 12937_2021_739_MOESM1_ESM.docx]

**Table S1.** Food sources of dietary fructose in urban and rural residents (g/d)

|  | Urban | | | |  | Rural | | | | | *p*-Value |
| --- | --- | --- | --- | --- | --- | --- | --- | --- | --- | --- | --- |
|  | Mean | P_25th_ | Median | P_75th_ |  | Mean | | P_25th_ | Median | P_75th_ |  |
| Grain and grain products | 0.95 | 0.37 | 0.65 | 1.19 |  | 1.15 | | 0.45 | 0.81 | 1.45 | <0.001 |
| Fruits and fruit products | 3.98 | 0.00 | 0.00 | 5.71 |  | 1.73 | | 0.00 | 0.00 | 1.10 | <0.001 |
| Vegetables and vegetable products | 2.90 | 1.46 | 2.45 | 3.81 |  | 2.71 | | 1.24 | 2.16 | 3.58 | <0.001 |
| Milk and milk products | 0.29 | 0.00 | 0.00 | 0.05 |  | 0.05 | | 0.00 | 0.00 | 0.00 | <0.001 |
| Meat, poultry, fish, and related products | 0.41 | 0.13 | 0.29 | 0.54 |  | 0.28 | | 0.03 | 0.17 | 0.41 | <0.001 |
| Eggs and egg products | 0.55 | 0.00 | 0.39 | 0.84 |  | 0.32 | | 0.00 | 0.00 | 0.52 | <0.001 |
| Legumes and legume products | 0.36 | 0.00 | 0.17 | 0.47 |  | 0.23 | | 0.00 | 0.00 | 0.26 | <0.001 |
| Nuts, seeds, and related products | 0.10 | 0.00 | 0.00 | 0.00 |  | 0.06 | | 0.00 | 0.00 | 0.00 | <0.001 |
| Sugars and sweets | 0.23 | 0.00 | 0.00 | 0.00 |  | 0.06 | | 0.00 | 0.00 | 0.00 | <0.001 |
| Nonalcoholic beverages | 0.16 | 0.00 | 0.00 | 0.00 |  | 0.10 | | 0.00 | 0.00 | 0.00 | <0.001 |
| Alcoholic beverages | 0.49 | 0.00 | 0.00 | 0.00 |  | 0.63 | | 0.00 | 0.00 | 0.00 | 0.308 |
| Snacks | 1.14 | 0.00 | 0.06 | 1.36 |  | 0.25 | 0.00 | | 0.00 | 0.00 | <0.001 |
| Miscellaneous foods | 0.06 | 0.00 | 0.00 | 0.02 |  | 0.04 | 0.00 | | 0.00 | 0.00 | <0.001 |

Abbreviation: P = percentile

**Table S2.** Stratified analysis of the association between dietary fructose intake and the odds of having MetS by physical activity in rural residents

|  | Dietary fructose intake | | | | *p*-Value |
| --- | --- | --- | --- | --- | --- |
|  | Q1 | Q2 | Q3 | Q4 |  |
| Physical activity |  |  |  |  |  |
| MetS, n (%) | 42 (34.4) | 57 (37.3) | 69 (34.9) | 75 (32.6) | 0.829 |
| Model 1 | 1.00 | 1.13 (0.69, 1.86) | 1.02 (0.63, 1.64) | 0.92 (0.58, 1.47) | 0.829 |
| Model 2 | 1.00 | 1.02 (0.61, 1.71) | 0.99 (0.60, 1.64) | 0.96 (0.57, 1.61) | 0.995 |
| Model 3 | 1.00 | 1.00 (0.55, 1.81) | 1.07 (0.60, 1.91) | 1.05 (0.58, 1.91) | 0.992 |
| Non- physical activity |  |  |  |  |  |
| MetS, n (%) | 724 (24.1) | 711 (24.1) | 737 (25.2) | 658 (22.8) | 0.192 |
| Model 1 | 1.00 | 1.00 (0.89, 1.12) | 1.06 (0.94, 1.20) | 0.93 (0.82, 1.05) | 0.192 |
| Model 2 | 1.00 | 1.03 (0.91, 1.16) | 1.13 (0.99, 1.28) | 1.02 (0.89, 1.17) | 0.217 |
| Model 3 | 1.00 | 1.02 (0.89, 1.18) | 1.13 (0.98, 1.31) | 1.02 (0.88, 1.19) | 0.282 |

Model 1: crude; Model 2: adjusted gender, age, education, marital status, smoking, alcohol, income, energy, protein, fat, carbohydrate, fiber, TC; Model 3: model 2 plus BMI.

**Table S3.** Stratified analysis of the association between dietary fructose intake and the odds of having MetS by gender

|  | Dietary fructose intake | | | | *p*-Value |
| --- | --- | --- | --- | --- | --- |
|  | Q1 | Q2 | Q3 | Q4 |  |
| **Urban** |  |  |  |  |  |
| Male |  |  |  |  |  |
| MetS, n (%) | 426 (30.7) | 509 (33.3) | 527 (37.1) | 512 (36.1) | **0.001** |
| Model 1 | 1.00 | 1.13 (0.96, 1.32) | 1.33 (1.14, 1.56) | 1.27 (1.09, 1.49) | **0.001** |
| Model 2 | 1.00 | 1.08 (0.92, 1.27) | 1.19 (1.01, 1.41) | 1.04 (0.87, 1.25) | 0.157 |
| Model 3 | 1.00 | 1.03 (0.86, 1.24) | 1.15 (0.95, 1.38) | 1.07 (0.87, 1.31) | 0.512 |
| Female |  |  |  |  |  |
| MetS, n (%) | 593 (31.6) | 573 (33.0) | 582 (31.6) | 515 (27.9) | **0.007** |
| Model 1 | 1.00 | 1.07 (0.93, 1.22) | 1.00 (0.87, 1.15) | 0.84 (0.73, 0.96) | **0.007** |
| Model 2 | 1.00 | 1.08 (0.94, 1.25) | 1.02 (0.88, 1.19) | 0.90 (0.77, 1.06) | 0.127 |
| Model 3 | 1.00 | 1.04 (0.89, 1.22) | 1.02 (0.86, 1.20) | 0.89 (0.75, 1.07) | 0.301 |
| **Rural** |  |  |  |  |  |
| Male |  |  |  |  |  |
| MetS, n (%) | 262 (20.4) | 319 (22.1) | 343 (23.0) | 362 (22.8) | 0.354 |
| Model 1 | 1.00 | 1.11 (0.92, 1.34) | 1.17 (0.97, 1.40) | 1.15 (0.96, 1.38) | 0.354 |
| Model 2 | 1.00 | 1.09 (0.90, 1.32) | 1.16 (0.95, 1.41) | 1.15 (0.94, 1.41) | 0.472 |
| Model 3 | 1.00 | 1.12 (0.90, 1.40) | 1.20 (0.96, 1.51) | 1.18 (0.93, 1.49) | 0.428 |
| Female |  |  |  |  |  |
| MetS, n (%) | 502 (27.5) | 453 (27.0) | 462 (28.5) | 370 (24.3) | 0.052 |
| Model 1 | 1.00 | 0.98 (0.84, 1.14) | 1.05 (0.91, 1.22) | 0.85 (0.73, 0.99) | 0.052 |
| Model 2 | 1.00 | 1.00 (0.85, 1.16) | 1.11 (0.95, 1.31) | 0.93 (0.78, 1.11) | 0.181 |
| Model 3 | 1.00 | 0.98 (0.82, 1.16) | 1.11 (0.93, 1.33) | 0.93 (0.76, 1.12) | 0.235 |

Model 1: crude; Model 2: adjusted gender, age, education, marital status, smoking, alcohol, physical activity, income, energy, protein, fat, carbohydrate, fiber, TC; Model 3: model 2 plus BMI.

**Table S4.** Stratified analysis of the association between dietary fructose intake and the odds of having MetS by smoking

|  | Dietary fructose intake | | | | *p*-Value |
| --- | --- | --- | --- | --- | --- |
|  | Q1 | Q2 | Q3 | Q4 |  |
| **Urban** |  |  |  |  |  |
| Smoking |  |  |  |  |  |
| MetS, n (%) | 229 (29.5) | 247 (29.2) | 263 (33.9) | 221 (33.2) | 0.091 |
| Model 1 | 1.00 | 0.99 (0.80, 1.22) | 1.23 (0.99, 1.52) | 1.19 (0.95, 1.49) | 0.091 |
| Model 2 | 1.00 | 0.96 (0.77, 1.20) | 1.14 (0.91, 1.43) | 1.03 (0.80, 1.33) | 0.466 |
| Model 3 | 1.00 | 0.98 (0.76, 1.26) | 1.10 (0.854, 1.43) | 1.00 (0.75, 1.33) | 0.780 |
| Ever/Never-smoking |  |  |  |  |  |
| MetS, n (%) | 790 (31.7) | 835 (34.5) | 846 (34.0) | 806 (31.0) | **0.021** |
| Model 1 | 1.00 | 1.13 (1.01, 1.27) | 1.11 (0.98, 1.25) | 0.97 (0.86, 1.09) | **0.021** |
| Model 2 | 1.00 | 1.12 (0.99, 1.26) | 1.09 (0.96, 1.23) | 0.94 (0.82, 1.08) | **0.023** |
| Model 3 | 1.00 | 1.06 (0.93, 1.22) | 1.07 (0.93, 1.23) | 0.96 (0.83, 1.12) | 0.339 |
| **Rural** |  |  |  |  |  |
| Smoking |  |  |  |  |  |
| MetS, n (%) | 159 (18.2) | 175 (19.3) | 205 (21.5) | 212 (20.6) | 0.301 |
| Model 1 | 1.00 | 1.08 (0.85, 1.37) | 1.23 (0.98, 1.56) | 1.17 (0.93, 1.47) | 0.301 |
| Model 2 | 1.00 | 1.09 (0.85, 1.39) | 1.24 (0.97, 1.59) | 1.18 (0.91, 1.53) | 0.367 |
| Model 3 | 1.00 | 1.09 (0.82, 1.44) | 1.32 (1.00, 1.76) | 1.18 (0.88, 1.58) | 0.247 |
| Ever/Never-smoking |  |  |  |  |  |
| MetS, n (%) | 607 (27.0) | 593 (27.0) | 601 (27.8) | 521 (25.0) | 0.201 |
| Model 1 | 1.00 | 1.00 (0.87, 1.14) | 1.04 (0.91, 1.19) | 0.90 (0.79, 1.03) | 0.201 |
| Model 2 | 1.00 | 1.01 (0.88, 1.16) | 1.10 (0.95, 1.26) | 0.97 (0.83, 1.13) | 0.328 |
| Model 3 | 1.00 | 1.01 (0.87, 1.18) | 1.09 (0.93, 1.28) | 0.98 (0.82, 1.16) | 0.519 |

Model 1: crude; Model 2: adjusted gender, age, education, marital status, alcohol, physical activity, income, energy, protein, fat, carbohydrate, fiber, TC; Model 3: model 2 plus BMI.

**Table S5.** Stratified analysis of the association between dietary fructose intake and the odds of having MetS by alcohol use

|  | Dietary fructose intake | | | | *p*-Value |
| --- | --- | --- | --- | --- | --- |
|  | Q1 | Q2 | Q3 | Q4 |  |
| **Urban** |  |  |  |  |  |
| Alcohol |  |  |  |  |  |
| MetS, n (%) | 249 (26.6) | 296 (29.5) | 360 (34.1) | 380 (31.9) | **0.002** |
| Model 1 | 1.00 | 1.15 (0.95, 1.41) | 1.43 (1.18, 1.74) | 1.29 (1.07, 1.56) | **0.002** |
| Model 2 | 1.00 | 1.13 (0.92, 1.38) | 1.38 (1.13, 1.69) | 1.23 (0.99, 1.52) | **0.017** |
| Model 3 | 1.00 | 1.07 (0.85, 1.35) | 1.35 (1.07, 1.71) | 1.24 (0.97, 1.58) | 0.047 |
| Ever/Never- alcohol |  |  |  |  |  |
| MetS, n (%) | 770 (33.1) | 786 (34.7) | 749 (33.9) | 647 (31.2) | 0.083 |
| Model 1 | 1.00 | 1.08 (0.95, 1.22) | 1.04 (0.92, 1.17) | 0.92 (0.81, 1.04) | 0.083 |
| Model 2 | 1.00 | 1.07 (0.94, 1.21) | 1.00 (0.88, 1.14) | 0.87 (0.76, 1.01) | 0.393 |
| Model 3 | 1.00 | 1.03 (0.89, 1.18) | 0.98 (0.85, 1.13) | 0.88 (0.75, 1.03) | 0.197 |
| **Rural** |  |  |  |  |  |
| Alcohol |  |  |  |  |  |
| MetS, n (%) | 148 (18.7) | 192 (20.0) | 226 (22.9) | 274 (22.0) | 0.115 |
| Model 1 | 1.00 | 1.09 (0.86, 1.38) | 1.29 (1.02, 1.63) | 1.23 (0.98, 1.54) | 0.115 |
| Model 2 | 1.00 | 1.05 (0.82, 1.34) | 1.22 (0.96, 1.57) | 1.13 (0.88, 1.45) | 0.385 |
| Model 3 | 1.00 | 1.06 (0.80, 1.40) | 1.25 (0.95, 1.65) | 1.14 (0.87, 1.51) | 0.406 |
| Ever/Never-alcohol |  |  |  |  |  |
| MetS, n (%) | 616 (26.5) | 580 (26.9) | 579 (27.2) | 458 (24.5) | 0.204 |
| Model 1 | 1.00 | 1.02 (0.89, 1.17) | 1.04 (0.91, 1.18) | 0.90 (0.78, 1.03) | 0.204 |
| Model 2 | 1.00 | 1.05 (0.91, 1.20) | 1.10 (0.96, 1.27) | 0.98 (0.84, 1.14) | 0.349 |
| Model 3 | 1.00 | 1.04 (0.89, 1.21) | 1.11 (0.95, 1.30) | 0.97 (0.82, 1.16) | 0.395 |

Model 1: crude; Model 2: adjusted gender, age, education, marital status, smoking, physical activity, income, energy, protein, fat, carbohydrate, fiber, TC; Model 3: model 2 plus BMI.
